# HLA-B*44 and the Bw4-80T motif are associated with poor outcome of relapse-preventive immunotherapy in acute myeloid leukemia

**DOI:** 10.1007/s00262-023-03506-3

**Published:** 2023-08-19

**Authors:** Hana Komic, Alexander Hallner, Brwa Ali Hussein, Chiara Badami, Anne Wöhr, Kristoffer Hellstrand, Elin Bernson, Fredrik B. Thorén

**Affiliations:** 1 TIMM Laboratory at Sahlgrenska Center for Cancer Research, University of Gothenburg, Gothenburg, Sweden; 2 Department of Medical Biochemistry and Cell Biology, Institute of Biomedicine, Sahlgrenska Academy, University of Gothenburg, Gothenburg, Sweden; 3 Department of Infectious Diseases, Institute of Biomedicine, Sahlgrenska Academy, University of Gothenburg, Gothenburg, Sweden; 4 Department of Obstetrics and Gynecology, Institute of Clinical Sciences, Sahlgrenska Academy, University of Gothenburg, Gothenburg, Sweden

**Keywords:** NK cells, KIR3DL1, HLA-B*44, AML, IL-2, Histamine

## Abstract

HLA-B alleles are associated with outcomes in various pathologies, including autoimmune diseases and malignancies. The encoded HLA-B proteins are pivotal in antigen presentation to cytotoxic T cells, and some variants containing a Bw4 motif also serve as ligands to the killer immunoglobulin-like receptors (KIR) 3DL1/S1 of NK cells. We investigated the potential impact of HLA-B genotypes on the efficacy of immunotherapy for relapse prevention in acute myeloid leukemia (AML). Seventy-eight non-transplanted AML patients receiving HDC/IL-2 in the post-consolidation phase were genotyped for HLA-B and KIR genes. HLA-B*44 heralded impaired LFS (leukemia-free survival) and overall survival (OS), but the negative association with outcome was not shared across alleles of the HLA-B44 supertype. Notably, HLA-B*44 is one of few HLA-B44 supertype alleles containing a Bw4 motif with a threonine at position 80, which typically results in weak binding to the inhibitory NK receptor, KIR3DL1. Accordingly, a strong interaction between KIR3DL1 and Bw4 was associated with superior LFS and OS (*p* = 0.014 and *p* = 0.027, respectively). KIR3DL1^**+**^ NK cells from 80 T-Bw4 donors showed significantly lower degranulation responses and cytokine responses than NK cells from 80I-Bw4 donors, suggesting impaired KIR3DL1-mediated education in 80 T-Bw4 subjects. We propose that presence of a strong KIR3DL1^+^–Bw4 interaction improves NK cell education and thus is advantageous in AML patients receiving HDC/IL-2 immunotherapy for relapse prevention.

**Supplementary Information:**

The online version contains supplementary material available at 10.1007/s00262-023-03506-3.

## Introduction

Acute myeloid leukemia (AML) is characterized by clonal expansion of immature myeloid cells in the bone marrow and peripheral blood [1]. Intensive induction chemotherapy yields high rates of complete remission (CR) but many patients relapse despite repeated courses of consolidation chemotherapy in the post-CR phase. Eligible patients may receive allogeneic stem cell transplants and older patients may benefit from post-consolidation chemotherapy for remission maintenance [2] but there remains a need for additional relapse-preventive therapies [3]. A phase III trial of 320 AML patients in remission showed that treatment with histamine dihydrochloride and interleukin-2 (HDC/IL-2) significantly improved leukemia-free survival (LFS) versus standard of care in patients below 60 years of age [4]. In this regimen, IL-2 activates natural killer (NK) and T cells, while HDC targets H_2_ receptors expressed by myeloid cells to inhibit the formation of immunosuppressive oxygen radicals [5–7].

Human leukocyte antigen (HLA) class I genes are highly polymorphic, and the encoded proteins have a key role in antigen presentation. Peptides derived from intracellular proteins bind to the groove of the HLA molecule and are presented to cytotoxic CD8^+^ T cells. Different HLA alleles that share capacity to present similar peptides are commonly grouped into nine HLA supertypes [8]. A particular neoantigen from malignant cells can preferentially be presented by HLA molecules of a specific supertype, leading to detection by specific T cells and the elimination of malignant cells. For example, HLA-B44 supertype molecules feature an electropositive binding pocket and preferentially display peptides with negatively charged amino acid anchors. Mutations causing glycine-to-glutamic acid substitutions are common in melanoma, and it was proposed that this contributes to the favorable outcome of HLA-B44 supertype patients in immune checkpoint blockade (ICB)-treated melanoma [9]. However, the beneficial impact of the B44 supertype was not observed in lung cancer [10], B cell lymphoma (DLBCL) [11] or uveal melanoma [12]. Conversely, the HLA-B62 supertype and the HLA-A03 allele have been associated with inferior outcome of ICB therapy [9, 13].

Like T cells, NK cells express a battery of receptors that recognize HLA molecules, such as the killer immunoglobulin-like receptors (KIRs). The *KIR* locus harbors a family of highly homologous and polymorphic genes, and the encoded receptors recognize specific HLA molecules. Thereby, NK cells will in general spare healthy cells with intact expression of HLA class I molecules, while cells with absent or reduced HLA class I expression will fail to generate sufficient inhibitory signals in NK cells and are killed in a process known as missing self-recognition. All HLA-C alleles serve as ligands to KIR2D receptors, while subsets of HLA-A and HLA-B alleles contain a Bw4 motif that ligates KIR3DL1. A dimorphism at HLA-B Bw4 position 80, with isoleucine (Bw4-80I) or threonine (Bw4-80 T) variants, has been reported to determine the affinity for KIR3DL1 [14]. Interestingly, such Bw4 variants were reported to be of clinical relevance in viral infections and malignancies [15–17]. Bw4 motifs are also present in a few HLA-A alleles, but it is still unclear to what extent these alleles contribute to KIR3DL1-mediated education and inhibition [18–20].

This study aimed at determining the potential impact of HLA-B genotypes on outcome of HDC/IL-2-based immunotherapy in AML. We observed that HLA-B*44, but not other members of the HLA-B44 supertype, was associated with distinctly poor LFS. Notably, the HLA-B*44 alleles encode a Bw4-80 T motif with low affinity for KIR3DL1. Individuals with this Bw4 variant harbored KIR3DL1^+^ NK cells with inferior degranulation and cytokine release responses to leukemic cells, which may contribute to the poor survival after AML immunotherapy.

## Materials and methods

### Patients

An open-label single-arm phase IV study (Re:Mission, NCT01347996) enrolled 84 (age 18–79) patients in first CR with de novo or secondary AML. Patients received ten 21-day courses of HDC (0.5 mg by subcutaneous injection twice daily) and IL-2 (16 400 IU/kg by subcutaneous injection twice daily) over a period of 18 months or until relapse/death. A primary objective of the study was to monitor quantitative and qualitative pharmacodynamics effects with focus on the phenotype and function of NK, T and myeloid cells. All patients gave written informed consent prior to enrollment and the trial was approved by the Ethical committees in all participating regions. This study was conducted according to the Declaration of Helsinki principles. Results from the trial have been previously published [21–24]. Detailed patient characteristics can be found in previously published papers [21, 22].

### Sampling of peripheral blood

Peripheral blood was collected before and after the first and third treatment cycles. Peripheral blood mononuclear cells (PBMCs) were isolated by density gradient centrifugation using Lymphoprep (Stemcell Technologies), cryopreserved at local sites and shipped on dry ice to the central laboratory at University of Gothenburg for analysis by flow cytometry. Samples with < 25% viability were excluded from any analyzes.

Leukopacks from healthy blood donors were obtained from Sahlgrenska University Hospital, and PBMCs were isolated by density gradient centrifugation using Lymphoprep (Stemcell Technologies) and cryopreserved in liquid nitrogen. Isolation of NK cells was performed by negative selection using human NK cell isolation kit (Miltenyi Biotec) in accordance with manufacturer’s instructions.

### DNA extraction and KIR/HLA genotyping

DNA was extracted from whole blood using a Roche MagNAPure 96 or Qiagen® DNeasy Blood & Tissue kit according to the manufacturer’s instructions. KIR3DL1 typing was performed using PCR primers described by Boudreau et al*.* to detect the major alleles *004 (null), *001, *002 (high), *013 (3DS1) and *005, *007 (low). [25]. Furthermore, flow cytometry was used to determine the KIR3DL1 expression level by staining cells with anti-CD3-APC-H7 (SK7), anti-CD14-APC-H7 (MϕP9), anti-CD16-BV786 (3G8), anti-CD56-BV711 (NCAM1), anti-CD57-BV605 (NK-1), anti-CD19-APC-H7 (SJ25C1; all from BD Biosciences), anti-NKG2C-Alexa Fluor 488 (134,591), anti-KIR2DL1 Alexa Fluor 700 (143211, both R&D Systems), anti-NKG2A-PE (Z199), anti-KIR2DL1/S1-Pe-Cy7 (EB6B), anti-KIR2DL2/L3/S2-Pe-Cy5.5 (GL 183; all from Beckman Coulter), anti-KIR3DL1-APC (DX9; BioLegend). Stained samples were analyzed on a five laser BD LSR Fortessa SORP instrument. Data was analyzed using FACSDiva (v.8.0.1 or later) and FlowJo (v.10.8.1 or later) software (BD Biosciences). The median frequency of KIR3DL1^+^ cells among CD16^+^CD56^+^ NK cells was 11% (range 7.71–56.5%). The HLA-B and -C allele genotype was determined using LABType SSO Class I Locus Typing Tests from One Lambda as described elsewhere [26]. Complementary KIR ligand typing for HLA-A Bw4 was performed using the Olerup SSP KIR HLA Ligand kit.

### HLA-B and HLA-Bw4 staining

To determine the expression levels of HLA-B and HLA-Bw4 in 80I/T versus 80 T groups of donors, cryopreserved PBMCs were stained with anti-CD3-FITC (HIT3a), anti-CD14-PE-Cy7 (M5E2), anti-CD56-BV711 (NCAM 16.2), anti-HLA-B-PE (YTH 76.3rMAb, all BD Biosciences), live-dead marker (near IR-APC-Cy7, Life Technologies) and anti-CD3-PerCP-Cy5.5 (SK7), anti-CD14-PE-Cy7 (M5E2), anti-CD56-BV711 (NCAM 16.2), anti-HLA-Bw4-FITC (REA274, Miltenyi Biotec), live-dead marker (near IR-APC-Cy7, Life Technologies). Analyzes were performed on a five laser BD LSR Fortessa SORP instrument and FlowJo software (v.10.8.1 or later) (BD Biosciences).

### Functional assays

NK cells were cultured overnight with 100 IU/ml of IL-2 (Proleukin, Novartis Pharmaceuticals) or non-stimulated (resting) in 0.1 million cells/well before addition of 0.1 million K562 cells/well. Anti-CD107a-BUV395 (H4A3, BD Biosciences) was added to measure the degranulation. After 4 h-incubation, cells were stained with anti-CD56-BV711 (NCAM 16.2, BD Biosciences), anti-NKG2A-PE (Z199), anti-KIR2DL1/S1-PE-Cy7 (EB6B), anti-KIR2DL2/L3/S2-PE-Cy5.5 (GL183; all Beckman Coulter) and anti-KIR3DL1-APC (DX9, Biolegend). Gating strategy is shown in Supplementary Fig. 1.

In intracellular cytokine assays, 50 000 NK cells/well were cultured and stimulated overnight, using same conditions as in degranulation assays. A five-hour co-culture with 50 000 K562 cells/well at 37 °C was performed, adding Brefeldin A (GolgiPlug, BD Biosciences) after one hour. The cells were stained with the same antibody panel as mentioned above, followed by fixation and permeabilization with BD Cytofix/Cytoperm (BD Biosciences). At the end, cells were stained with anti-IFNγ-BUV395 (B27) and anti-TNFα-AF488 (Mab 11; both BD Biosciences).

Stained samples were analyzed using a five laser BD LSR Fortessa SORP instrument. Data was analyzed using FACSDiva (v.8.0.1 or later) and FlowJo (v.10.8.1) software (BD Biosciences).

### Statistical analyses

All statistical analyses were performed in Prism v. 9 or later (GraphPad Software). The logrank test (Mantel-Cox) was used to compare survival between patient groups. Incidence of relapse was calculated using Chi square test. The impact of HLA-B and HLA-A alleles on LFS was analyzed by univariate and multivariate Cox regression analyses using IBM SPSS Statistics (v. 29). Mann–Whitney test was used to compare responses in degranulation and intracellular cytokine staining assays between groups.

## Results

### HLA-B*44 associates with inferior leukemia-free survival

Seventy-six patients from the Re:Mission trial were genotyped for HLA-B alleles. Out of 24 detected alleles, the most common was B*44 (detected in 28% of patients, allele frequency = 0.15), followed by B*35 (21%, 0.11), B*7 (20%, 0.11) and B*8 (20%, 0.11). The other alleles detected in > 10% of patients were B*51 and B*40 (Table 1).

**Table 1:** HLA-B allele frequency and impact on LFS

Allele	Detected in% patients	Allele frequency	Univariate Cox regression analysis of LFS	Multivariate Cox regression analysis of LFS
B*07	20	0.112	0.055	0.517
B*08	20	0.105	0.379	0.971
B*35	21	0.112	0.993	0.245
B*40	14	0.072	0.562	0.102
B*44	28	0.151	< 0.001	0.005
B*51	17	0.086	0.524	0.836

**Fig. 1:**
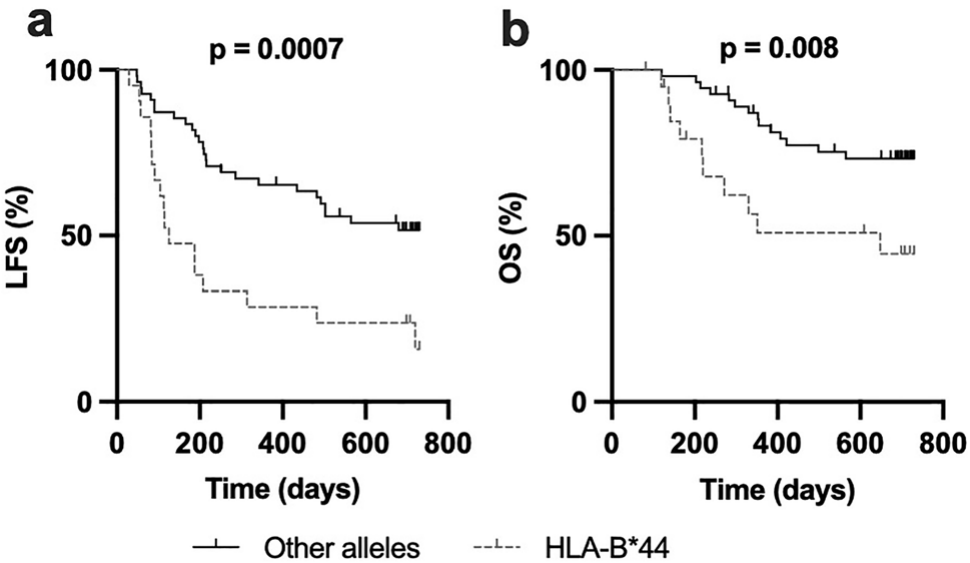
The HLA*B44 allele is associated with significantly inferior LFS and OS. **a** Leukemia-free survival and **b** Overall survival for patients with at least one HLA*B44 allele (n = 21) compared with patients lacking HLA-B*44 alleles (n = 55). (Log rank test for trend)

To determine whether HLA-B allotypes had an impact on clinical outcome, we performed univariate and multivariate Cox regression analyses of leukemia-free survival (LFS) for all alleles with an allele frequency of > 10% in the cohort. As shown in Table 1, patients with at least one HLA-B*44 allele showed significantly inferior LFS. Accordingly, Kaplan–Meier survival analyses showed that patients harboring an HLA-B*44 (n = 21) allele had inferior LFS and overall survival (OS) when compared to patients with other alleles (n = 55) (Fig. 1a, b; *p* = 0.0007 and *p* = 0.008, respectively).

**Fig. 2:**
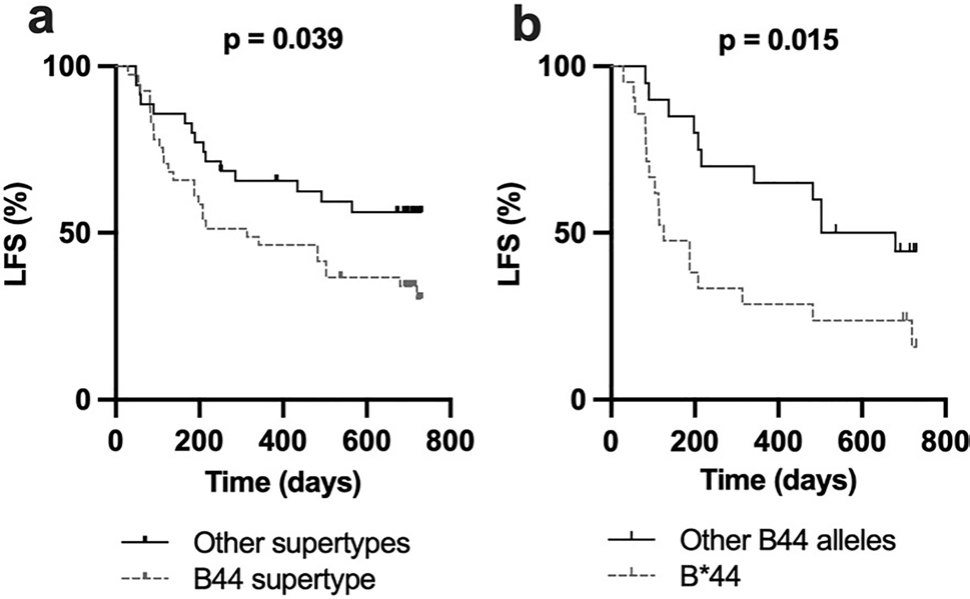
Inferior survival in the HLA-B44 supertype is driven by the HLA-B*44 allele **a** Leukemia-free survival of patients with alleles from the HLA-B44 supertype versus patients with alleles from any other HLA-B supertype (B07, B27, B58 and B62), **b** Leukemia-free survival among patients having at least one B*44 allele versus patients carrying any other non-HLA-B*44 allele of the B44 supertype. (Log rank test for trend)

Some HLA-B alleles display a similar capacity to bind and present peptides, and such alleles are thus commonly grouped into supertypes [27]. Survival analyses comparing the B44 supertype alleles (HLA-B*18, *37, *40, *41, *44, *47, *49 and *50) (n = 41) with all other B supertypes (n = 35) showed significantly superior LFS for patients who did not carry any B44 supertype alleles (*p* < 0.05; Fig. 2a). However, the effect of the B44 supertype on clinical outcome was clearly driven by HLA-B*44 as LFS was significantly higher in B*44 allele carriers (n = 21) than in non-B*44 allele carriers (n = 20) within the HLA-B44 supertype (Fig. 2b).

### Patients with Bw4-80 T have inferior clinical outcome

**Fig. 3:**
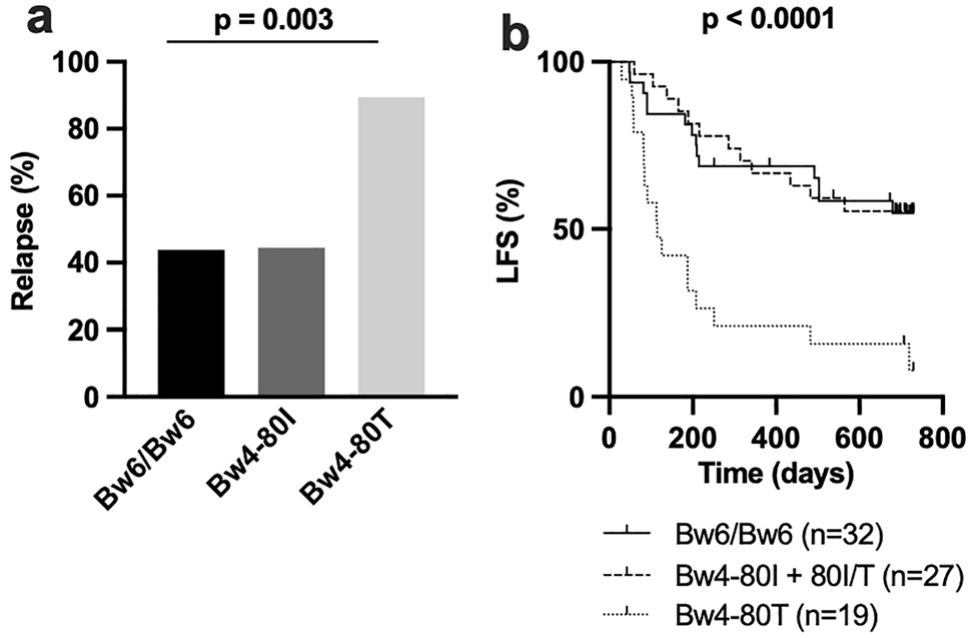
The Bw4 binding motif impacts survival outcome. **a** Relapse rates and **b** Kaplan–Meier analysis of patients with only a Bw4-80 T motif (Bw4-80 T/Bw4-80 T or Bw4-80 T/Bw6, n = 19), at least one Bw4-80I motif (Bw4-80I/Bw4-80I, Bw4-80I/Bw4-80 T or Bw4-80I/Bw6, n = 27) or Bw6/Bw6 (n = 32). (Log rank test for trend)

As stated above, HLA supertypes are groups of HLA alleles that share peptide binding features that allow them to present similar antigens to T cells. The finding that not all alleles within the B44 supertype share the negative association with LFS made us hypothesize that this discrepancy might be related to differential capacity of the encoded HLA-B molecules to interact with NK cell receptors. Although recent studies have demonstrated that some NK cell receptors may display peptide specificity [28], HLA interactions with NK cell receptors are typically less dependent on the presented peptide. Furthermore, NK cells are reportedly important for clinical outcome of HDC/IL-2 immunotherapy in AML [21, 22, 26, 29, 30]. HLA-B molecules contain either a Bw6 motif that does not interact with NK cell receptors, or one of two different Bw4 motifs, Bw4-80I and Bw4-80 T, which are recognized by the NK cell receptor KIR3DL1 [31]. Interestingly, data from the Immune polymorphism database showed that there are large differences within the B44 supertype regarding presence of Bw4 or Bw6 motifs [18]. HLA-B*37, HLA-B*44, and HLA-B*47 alleles harbor the Bw4-80 T motif, the HLA-B*49 allele contains a Bw4-80I motif, while the remaining major HLA-B44 supertype alleles detected encode the Bw6 motif. Thus, we next tested if relapse incidence was associated with Bw4 and Bw6 motifs. We speculated that having only one 80I allele is sufficient to enable education, as HLA-B and HLA-B Bw4 expression is much higher in 80I/T donors compared to 80 T donors (Supplementary Fig. 2). As shown in Fig. 3a patients with at least one Bw4-80 T allele and no 80I allele had higher relapse rates (Chi-square test, *p* = 0.003). Furthermore, these patients had a significantly inferior LFS (*p* < 0.0001) compared to 80I and Bw6 groups (Fig. 3b). Similar results were obtained if 80I/T patients were excluded from the analysis (*p* < 0.0001), or alternatively included to 80 T group (*p* = 0.001) (Supplementary Fig. 3a, b).

A few HLA-A alleles contain a Bw4 motif but it is still unclear to what extent these alleles contribute to KIR3DL1-mediated education and inhibition. In our patient cohort, HLA-A Bw4 did not show significant effect on LFS in 80I and 80 T groups (*p* = 0.35 and *p* = 0.71, respectively) (Supplementary Fig. 4). Additionally, to address to what extent HLA-A Bw4 presence affects the impact of HLA-B Bw4 alleles on the LFS, we performed univariate and multivariate Cox regression analyses, using HLA-A Bw4 presence/absence in combination with B*44 allele and HLA-B Bw4 motif as covariates, respectively. The findings regarding B*44 and B-80I versus 80 T remained highly significant (Supplementary Tables 1 and 2).

### Strong interacting 3DL1/HLA-B Bw4 pair favors leukemia-free survival

**Fig. 4:**
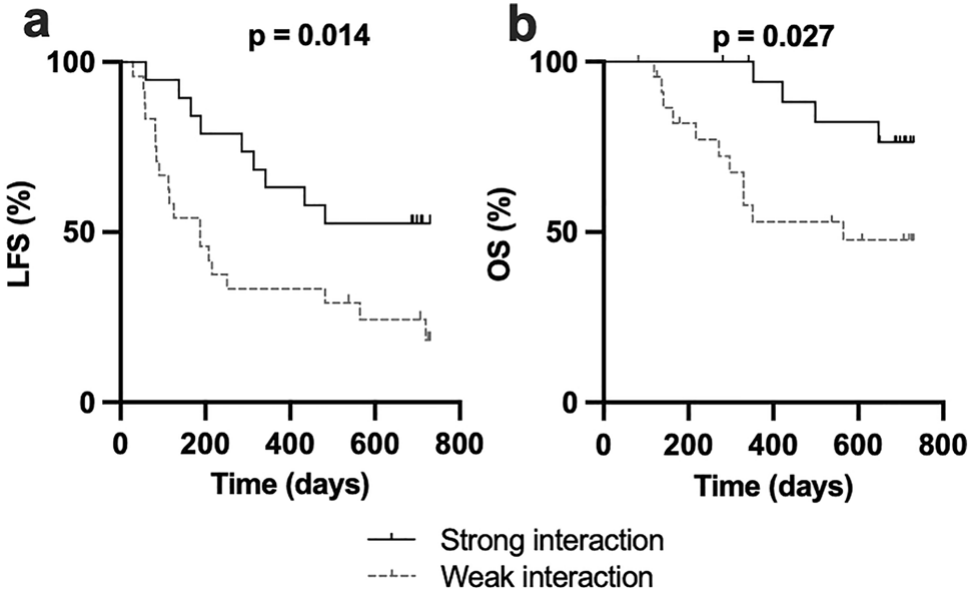
Weak 3DL1–Bw4 interaction is associated with inferior survival. **a** Leukemia -free survival and **b** Overall survival of patients with a strong interacting 3DL1–Bw4 pair (at least one KIR3DL1-high allele without low alleles together with a Bw4-80I allele) or weak interacting pair (KIR3DL1-low together with either of the Bw4 motifs) (Log rank test for trend)

Different combinations of KIR3DL1 and Bw4-containing HLA-B alleles result in 3DL1/HLA-B Bw4 interactions of varying affinity. KIR3DL1 alleles are divided into subgroups based on surface expression and function, and the subtypes KIR3DL1-high or KIR3DL1-low bind HLA-B Bw4-80I and 80 T with different affinity. We thus genotyped all HLA-B Bw4^+^ patients for KIR3DL1 alleles and grouped them into either high or low allele, using classification proposed by Boudreau et al. [32]. Strong KIR3DL1/HLA-B Bw4 interaction was defined by presence of at least one KIR3DL1-high allele without low alleles along with an HLA-B Bw4-80I, while low KIR3DL1 receptor in combination with either of the two HLA-B Bw4 motifs was classified as weak interaction. Two patients with *013/*004 (3DS1/null) allele combinations were excluded from the interaction figure. As shown in Fig. 4a, b, patients with a strong interacting pair (n = 19) showed significantly improved LFS and OS compared to patients with a weak interacting pair (n = 24). Similar results were obtained when KIR3DL1-high patients were defined by factual expression as determined by flow cytometry (Supplementary Fig. 5a, b).

### KIR3DL1 education

**Fig. 5:**
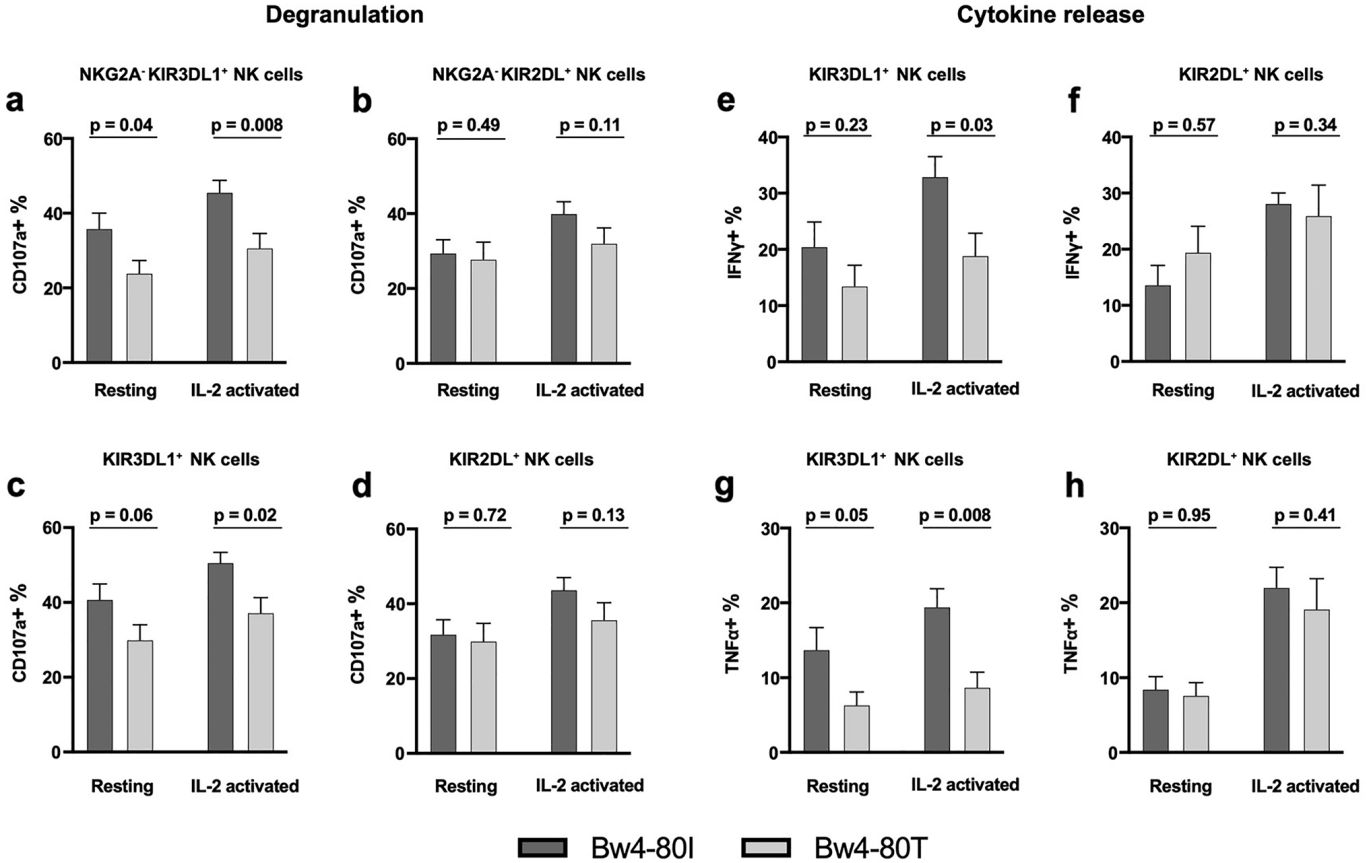
Degranulation assay in resting and IL-2 activated NK cells (a–d). **a** NKG2A-KIR3DL1sp cells, **b** NKG2A^−^KIR2D^+^ cells, **c** KIR3DL1^+^ cells, **d** KIR2D^+^ cells, (n = 19). **Cytokine release e–h. e** IFN-g production in KIR3DL1^+^ cells, **f** KIR2D^+^ cells, **g** TNF-a in 3DL1^+^ cells, **h** KIR2D.^+^ cells, (n = 13). Error bars represent SEM and Mann–Whitney test was used for statistical analyses. The mean degranulation response without K562 cells in absence of IL-2 was 1.6% (80I) and 1.0% (80 T), and 1.7% (80I) and 0.7% (80 T) in presence of IL-2. Corresponding results in the absence of K562 cells in intracellular cytokine release assays were consistently < 1% (data not shown)

To address the possible mechanisms underlying the inferior outcome for Bw4-80 T patients, we first determined the education status and ability to mount a missing-self response in KIR3DL1-single positive (sp) NK cells. In agreement with previous reports [33] there were significant differences between KIR3DL1^+^ NK cells from 80 T to 80I individuals in degranulation assays towards HLA-negative, NK cell-sensitive K562 cells. In the presence or absence of IL-2 pre-stimulation, the KIR3DL1^+^ NK cells from Bw4-80I donors degranulated significantly more than KIR3DL1^+^ NK cells from Bw4-80 T donors. Importantly, no such difference was seen when comparing NK cells educated via KIR2DL receptors in the same patients, suggesting that the observed difference was due to enhanced KIR3DL1 education in 80I individuals (Fig. 5a, b). The vast majority of NK cells from AML patients in the remission cohort were NKG2A^+^ cells. Accordingly, we tested and confirmed that the enhanced degranulation response was also seen in the corresponding NKG2A^+^KIR3DL1sp NK cells but not in NKG2A^+^KIR2DL^+^KIR3DL1^−^ cells (Fig. 5c, d). A similar pattern of increased NK cell responses to K562 cells within the Bw4-80I group was observed for IFNγ and TNFα production in KIR3DL1 cells but not in KIR2DL cells (Fig. 5f–h, Supplementary Fig. 6).

## Discussion

HLA-B is a highly polymorphic molecule implicated in immune cell recognition of malignant cells, and HLA-B genotypes have been linked to disease severity and treatment outcome in various pathologies. In this study, we set out to determine to what extent HLA-B genotypes are associated with treatment outcome in AML patients receiving immunotherapy for relapse prevention. The most striking finding in our study was that patients carrying B*44 alleles showed significantly inferior LFS and OS after HDC/IL-2 treatment. In contrast to these findings, ICB-treated melanoma patients with HLA-B44 supertype alleles reportedly show prolonged survival [9]. In that study it was proposed that the B44 supertype encompasses an electropositive binding pocket to preferentially display peptides with negatively charged amino acid anchors, which are common among melanoma neoantigens [9].

In our study the association with outcome appeared to be specific for the B*44 alleles as patients carrying HLA-B*44 alleles showed inferior outcome as compared to patients carrying non-HLA-B*44 alleles. Supertypes comprise HLA alleles that share peptide preferences, but the ability to serve as a KIR3DL1 ligand may vary within one HLA-B supertype. Within the B44 supertype, HLA B*37, B*44 and B*47 contain the Bw4-80 T epitope, which is a weak ligand to KIR3DL1 while other alleles encode either Bw6 or Bw4-80I, with the latter typically showing high affinity to KIR3DL1 [14, 18, 34, 35]. Intuitively, a weak inhibitory interaction between NK cells and leukemic targets should be advantageous and favor NK cell cytotoxicity. However, a weak interaction also means that the homeostatic inhibitory signaling to circulating NK cells via KIR3DL1 is low. These signals set the functional threshold for NK cells in a process known as education [36]. The more inhibitory input the NK cell receives at steady-state, the more vigorously the cell can respond to a target with down-regulated MHC class I expression. Accordingly, our data indicated that individuals carrying HLA-B Bw4-80 T harbored less functional single-positive KIR3DL1^+^ NK cells than donors with Bw4-80I, which is in agreement with previous reports [33]. Notably, it was reported that AML blasts display lower expression of HLA-Bw4 antigens than cells from healthy volunteers [37]. It is thus conceivable that NK cells can exert missing-self cytotoxicity against AML blasts, and that the response may be stronger if these anti-leukemic cells are highly educated via KIR3DL1 interactions with the high-affinity Bw4-80I ligands.

In line with this reasoning, studies of HIV infection show that NK cells from individuals with strong educating KIR3DL1–Bw4 interactions mount more efficient cytotoxic responses to HIV-infected cells [19] and that such individuals are less likely to progress to AIDS [17]. Furthermore, in uveal melanoma, which is a rare malignancy in which NK cells are believed to be key mediators in preventing metastasis formation, HLA-B*44 allele carriers were reported to have a significantly inferior survival [12]. The interaction between KIR3DL1 and Bw4 variants has also been the focus of multiple studies of allogeneic stem cell transplantation in AML. Some studies have, in contrast to the studies above, suggested reduced relapse risk and a survival benefit in patients receiving grafts resulting in low KIR3DL1 inhibition [32, 38] but a large retrospective study of over 2000 transplanted patients did not confirm these results [39]. More studies are thus warranted to clarify the role of KIR3DL1–Bw4 interactions in transplanted and non-transplanted AML.

In summary, we show that expression of host HLA-B*44 is associated with inferior survival in AML patients receiving immunotherapy for relapse prevention. We hypothesize that this HLA-B variant, which is a weak ligand to the NK cell receptor, KIR3DL1, results in hypofunctional KIR3DL1^+^ NK cells that fail to mount strong anti-leukemic responses.

### Supplementary Information

Below is the link to the electronic supplementary material.

Supplementary Fig. 1 Gating strategy for the functional assays (JPG 718 KB)

Supplementary Fig. 2 FACS staining results for healthy control PBMCs (a-b). One 80I allele is sufficient to induce a high expression of (a) HLA-B and (b) HLA-Bw4 when comparing Bw4-80I/T donors to Bw4-80T B*44 donors. Error bars represent SEM (JPG 58 KB)

Supplementary Fig. 3 Bw4-80I/80T patients have an intermediary outcome (a-b). Results are not strongly affected if they are (a) excluded from the analysis or (b) placed in Bw4-80T group (Log rank test for trend) (JPG 82 KB)

Supplementary Fig. 4 The presence of HLA-A Bw4 alleles does not substantially affect the impact of HLA-B Bw4 alleles on LFS (a-b). Within (a) Bw4-80I or (b) Bw4-80T group (JPG 65 KB)

Supplementary Fig. 5 Weak KIR3DL1 – Bw4 interaction is associated with inferior survival (ab) (a) KIR3DL1 measured by flow cytometry shows a clear difference between high and low expression, (b) survival plot (Log rank test for trend) (JPG 63 KB)

Supplementary Fig. 6 Cytokine response, polyfunctional data (a-b). (a) Resting cells and (b) IL-2 activated cells. Error bars represent SEM and Mann-Whitney test used for statistical analyses (JPG 73 KB)

Supplementary file7 (DOCX 13 KB)

Supplementary file8 (DOCX 13 KB)
