# Supplementary figures and images for: HLA-B*44 and the Bw4-80T motif are associated with poor outcome of relapse-preventive immunotherapy in acute myeloid leukemia
Source: Cancer Immunol Immunother. 2023 Aug 19;72(11):3559–66. doi: 10.1007/s00262-023-03506-3 (PMC10576699; doi:10.1007/s00262-023-03506-3)

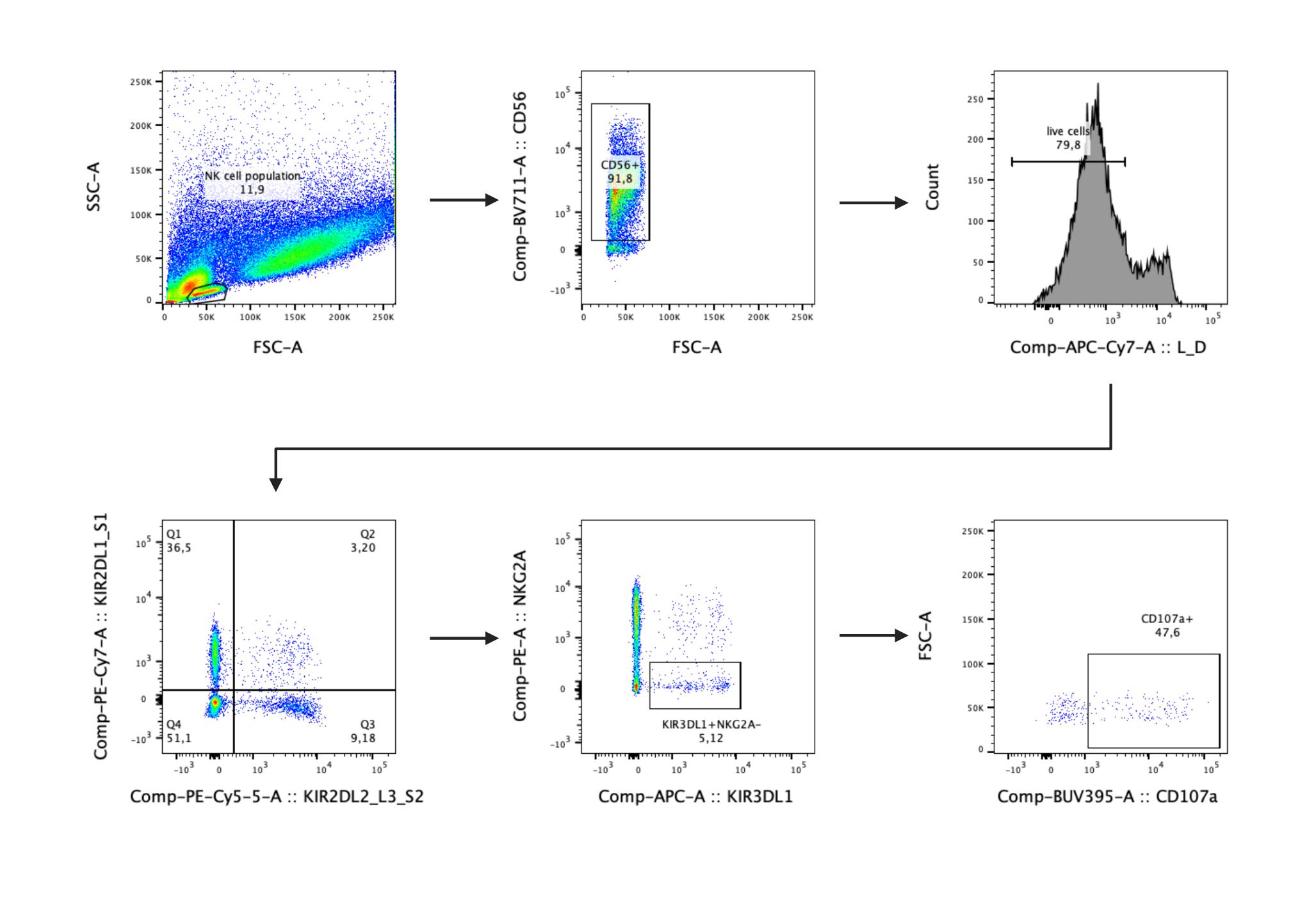

Supplement: Supplementary file 1 — Supplementary Fig. 1 Gating strategy for the functional assays (JPG 718 KB) [file 262_2023_3506_MOESM1_ESM.jpg]

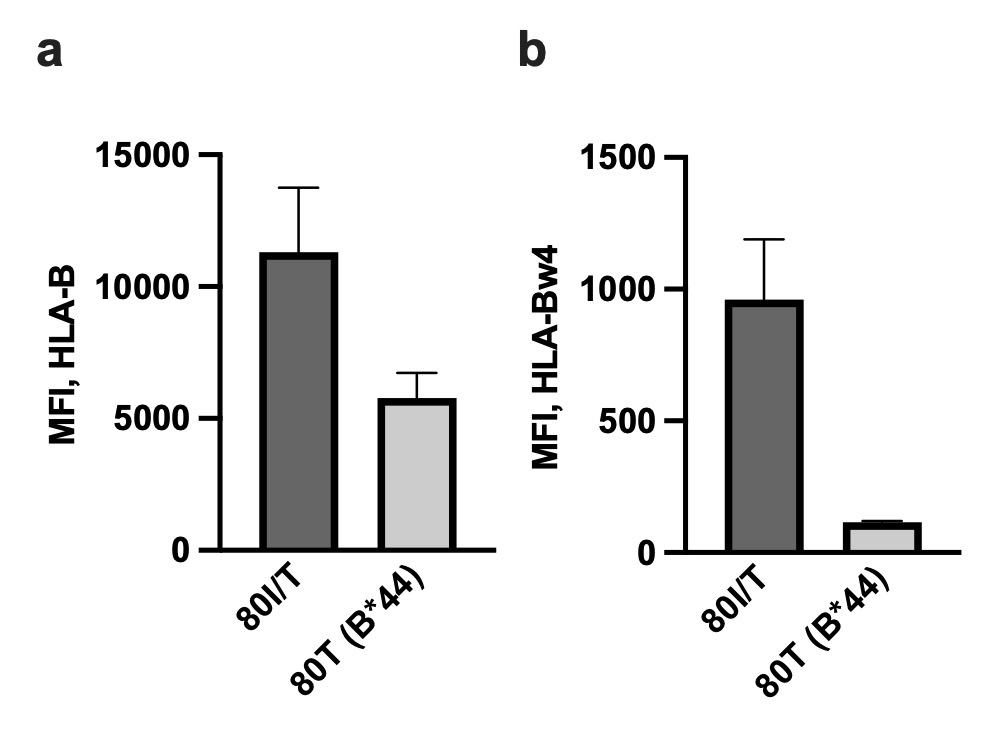

Supplement: Supplementary file 2 — Supplementary Fig. 2 FACS staining results for healthy control PBMCs (a-b). One 80I allele is sufficient to induce a high expression of (a) HLA-B and (b) HLA-Bw4 when comparing Bw4-80I/T donors to Bw4-80T B*44 donors. Error bars represent SEM (JPG 58 KB) [file 262_2023_3506_MOESM2_ESM.jpg]

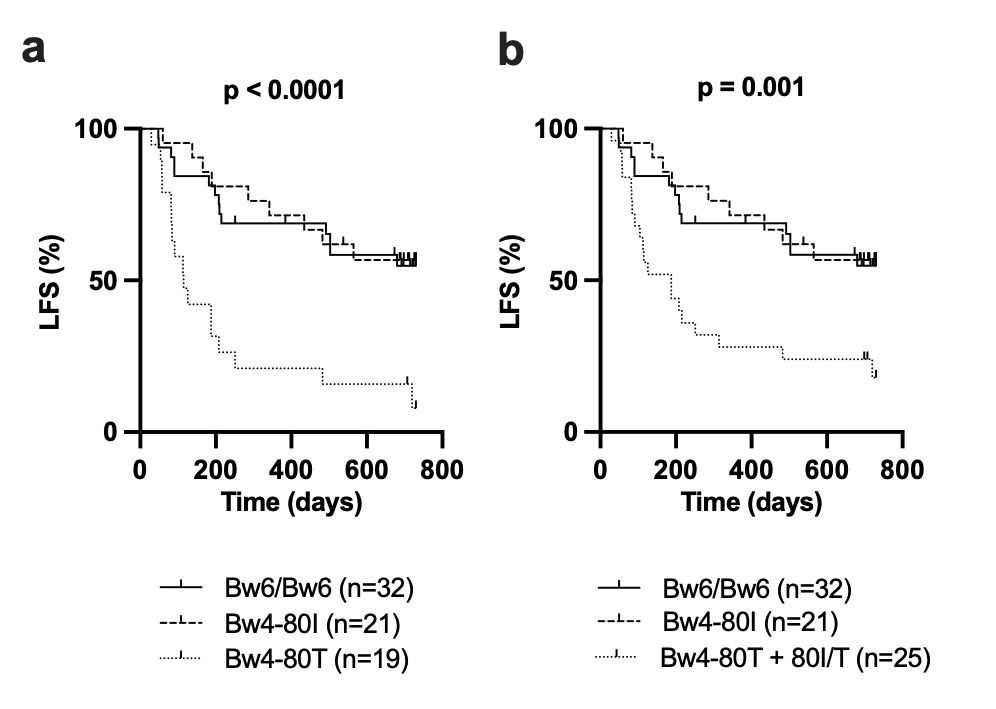

Supplement: Supplementary file 3 — Supplementary Fig. 3 Bw4-80I/80T patients have an intermediary outcome (a-b). Results are not strongly affected if they are (a) excluded from the analysis or (b) placed in Bw4-80T group (Log rank test for trend) (JPG 82 KB) [file 262_2023_3506_MOESM3_ESM.jpg]

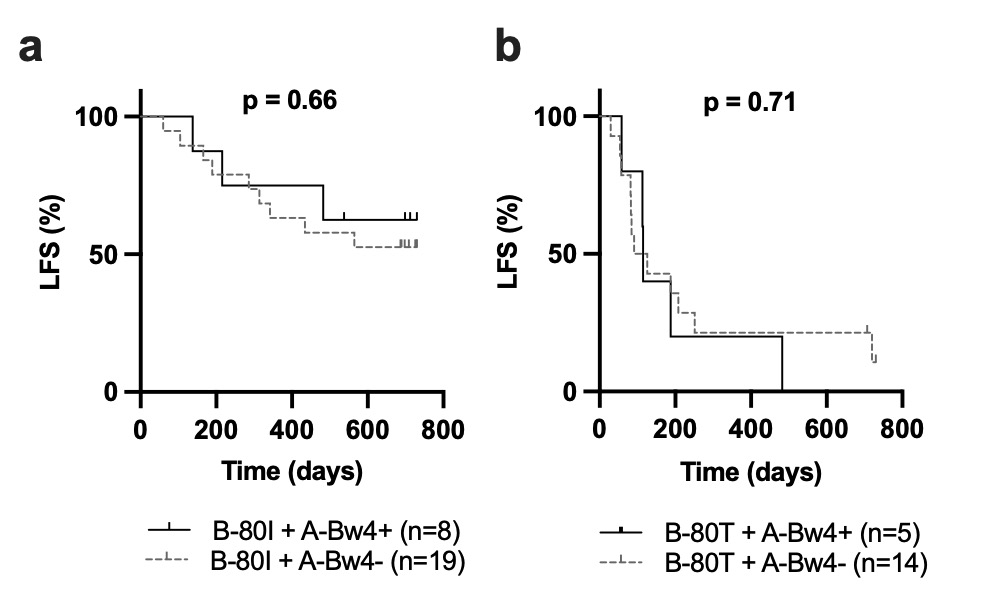

Supplement: Supplementary file 4 — Supplementary Fig. 4 The presence of HLA-A Bw4 alleles does not substantially affect the impact of HLA-B Bw4 alleles on LFS (a-b). Within (a) Bw4-80I or (b) Bw4-80T group (JPG 65 KB) [file 262_2023_3506_MOESM4_ESM.jpg]

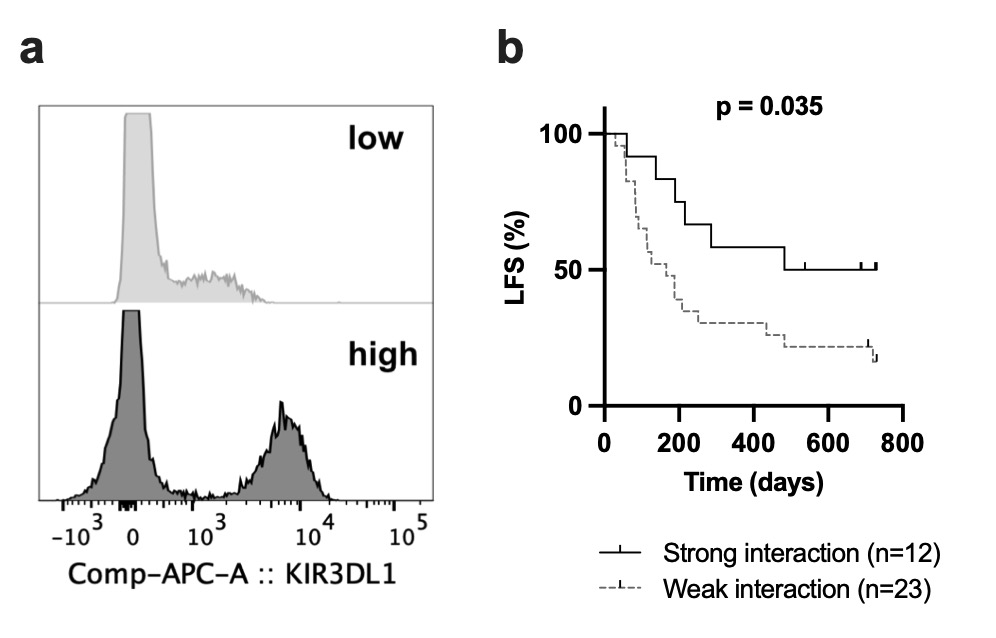

Supplement: Supplementary file 5 — Supplementary Fig. 5 Weak KIR3DL1 – Bw4 interaction is associated with inferior survival (ab) (a) KIR3DL1 measured by flow cytometry shows a clear difference between high and low expression, (b) survival plot (Log rank test for trend) (JPG 63 KB) [file 262_2023_3506_MOESM5_ESM.jpg]

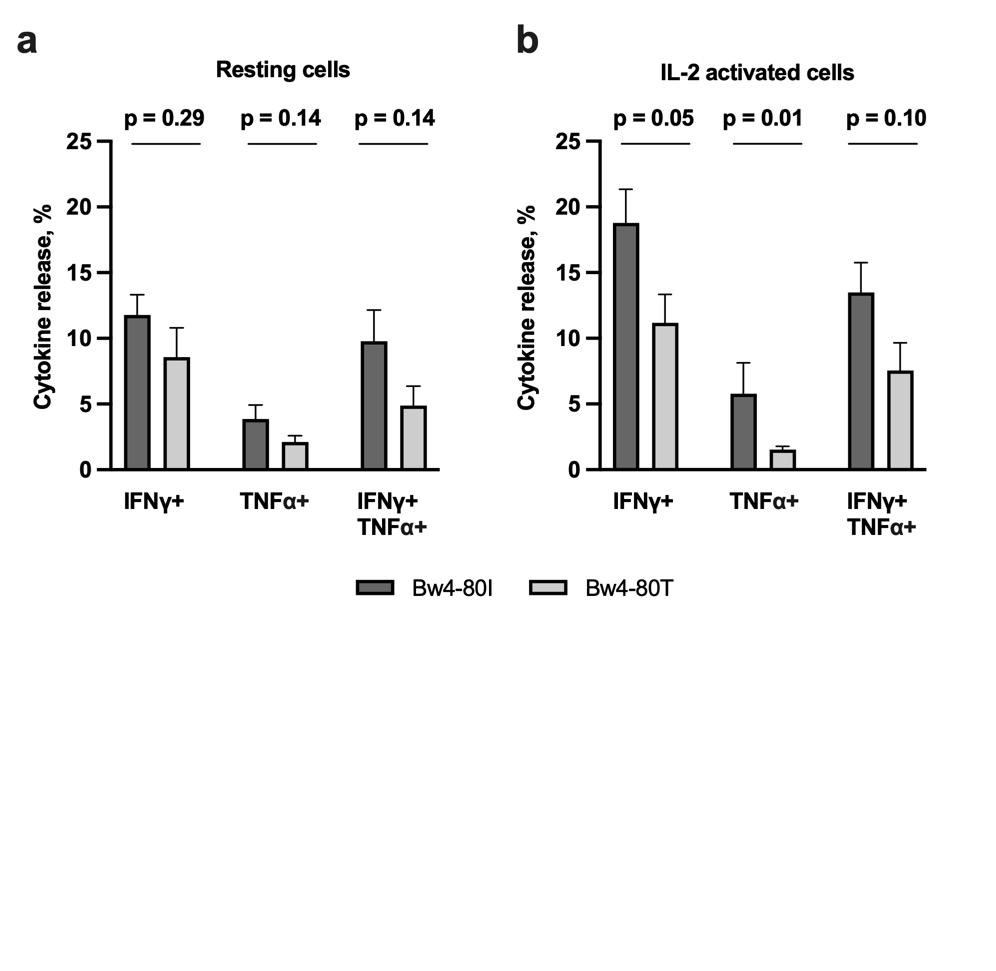

Supplement: Supplementary file 6 — Supplementary Fig. 6 Cytokine response, polyfunctional data (a-b). (a) Resting cells and (b) IL-2 activated cells. Error bars represent SEM and Mann-Whitney test used for statistical analyses (JPG 73 KB) [file 262_2023_3506_MOESM6_ESM.jpg]
