# Supplementary material for: HLA-B*44 and the Bw4-80T motif are associated with poor outcome of relapse-preventive immunotherapy in acute myeloid leukemia
Source: Cancer Immunol Immunother. 2023 Aug 19;72(11):3559–66. doi: 10.1007/s00262-023-03506-3 (PMC10576699; doi:10.1007/s00262-023-03506-3)
Supplement: Supplementary file 7 — Supplementary file7 (DOCX 13 KB) [file 262_2023_3506_MOESM7_ESM.docx]

**Supplementary Table 1.** Univariate and multivariate Cox regression analysis of LFS using presence/absence of B*44 allele and HLA-A Bw4 as covariates.

| Covariates | **Univariate analysis** | | | **Multivariate analysis** | | |
| --- | --- | --- | --- | --- | --- | --- |
|  | **HR** | **95% CI** | ***p* value** | **HR** | **95% CI** | ***p* value** |
| B*44 allele | 2.92 | 1.57-5.42 | <0.001 | 2.92 | 1.57-5.42 | <0.001 |
| HLA-A Bw4 | 1.09 | 0.58-2.05 | 0.781 | 1.03 | 0.55-1.92 | 0.938 |
