# Supplementary material for: HLA-B*44 and the Bw4-80T motif are associated with poor outcome of relapse-preventive immunotherapy in acute myeloid leukemia
Source: Cancer Immunol Immunother. 2023 Aug 19;72(11):3559–66. doi: 10.1007/s00262-023-03506-3 (PMC10576699; doi:10.1007/s00262-023-03506-3)
Supplement: Supplementary file 8 — Supplementary file8 (DOCX 13 KB) [file 262_2023_3506_MOESM8_ESM.docx]

**Supplementary Table 2.** Univariate and multivariate Cox regression analysis of LFS using HLA-B Bw4 motif and presence/absence of HLA-A Bw4 as covariates.

| Covariates | **Univariate analysis** | | | **Multivariate analysis** | | |
| --- | --- | --- | --- | --- | --- | --- |
|  | **HR** | **95% CI** | ***p* value** | **HR** | **95% CI** | ***p* value** |
| HLA-B Bw4 (80I or 80T) | 0.24 | 0.11-0.51 | <0.001 | 0.24 | 0.11-0.51 | <0.001 |
| HLA-A Bw4 | 0.92 | 0.41-2.07 | 0.834 | 1.01 | 0.44-2.29 | 0.987 |
